# Quality of life among hemodialysis patients in a referral center in Kathmandu: A mixed method study

**DOI:** 10.1371/journal.pone.0335990

**Published:** 2025-11-03

**Authors:** Shanti Gurung, Nishchal Devkota

**Affiliations:** Department of Public Health, Modern Technical College, Lalitpur, Nepal; The University of the West Indies, JAMAICA

## Abstract

**Introduction:**

Chronic kidney disease (CKD) affects about 10% of people worldwide, with rising cases in Nepal. Late diagnosis and limited healthcare access worsen patients’ quality of life (QoL), causing physical symptoms and emotional distress. Financial strain and travel difficulties further burden Nepalese patients on dialysis. While medical treatments address physical issues, mental and social well-being remain overlooked. This mixed-method study explores factors affecting QoL and patients lived experiences, aiming to improve patient-centered care in Nepal.

**Methods:**

This convergent parallel mixed-method study was conducted at the National Kidney Center in Kathmandu, Nepal, involving adult hemodialysis patients. A quantitative survey of 260 participants was conducted, and 15 purposively selected patients took part in an in-depth qualitative interview until data saturation. Data were collected simultaneously during dialysis sessions using validated Nepali versions of the KDQOL-36 and socio-demographic questionnaires. Quantitative data were analyzed with descriptive and non-parametric tests in SPSS, while qualitative data were transcribed, translated, and thematically coded based on KDQOL domains using a phenomenological approach. Ethical approval was obtained, and informed consent was secured from all participants, ensuring confidentiality and voluntary participation.

**Results:**

This study examined CKD patients on hemodialysis. Most were male (64.2%), married (76.5%), and in advanced stage of CKD. Treatment burden was reported by 74.2%. Median symptom score was 66.67; physical and mental health median scores were 32.62 and 39.40. Males and those with lower education reported higher burden. Socio-economic status and financial strain significantly affected outcomes. No significant differences were found by marital status, diet, or CKD duration. Patients on hemodialysis experienced significant symptom burden, including fatigue, weight loss, pain, and mobility issues, leading to dependency and emotional distress. Mental health challenges, such as anxiety, depression, and fear of the future, were common, especially among those with financial strain. Family and social support, acceptance, and coping strategies helped some maintain resilience. Socio-economic status was strongly linked to symptom severity and quality of life, emphasizing the need for holistic support in care.

**Conclusion:**

For hemodialysis patients in Nepal, managing physical symptoms is only part of the struggle; emotional distress, financial pressure, and loss of independence weigh more heavily. These burdens are intensified by limited mental health support, financial security, and access to decentralized care. Addressing these challenges requires a compassionate, patient-centered approach that goes beyond survival; supporting dignity, emotional well-being, and a better quality of daily life.

## Introduction

Chronic kidney disease (CKD) is a serious, long-term condition where the kidneys slowly lose their ability to function. It is defined by a glomerular filtration rate (GFR) below 60 mL/min/1.73m^2^ for three months or more, regardless of the cause [1,2]. As CKD progresses, it can lead to end-stage renal disease (ESRD), where kidney failure becomes life-threatening and patients need dialysis or kidney transplantation to survive Collaboration [3,4].

Globally, CKD affects around 10% of the population and is a leading cause of death and disability [3,5]. The burden is even higher in developing countries like Nepal, where healthcare resources are limited and most people cannot afford lifelong treatments like dialysis [6]. In Nepal, CKD affected over 1.8 million people in 2019, with the prevalence increasing significantly from 5,979 per 100,000 in 1990–7,634 per 100,000 in 2019 [7].

One major challenge with CKD is that it often goes unnoticed in the early stages. Many patients are diagnosed only after serious symptoms appear, by which time kidney damage is advanced and irreversible. At this stage, patients often experience a significant decline in emotional well-being and quality of life, particularly among older adults undergoing hemodialysis [8]. Once patients begin dialysis, they commonly experience fatigue, pain, poor sleep, and other physical problems that make daily life difficult [9–11].

However, the impact of CKD extends beyond physical symptoms. Many patients also suffer from depression, anxiety, and emotional stress due to lifestyle changes, financial pressures, and fear of worsening health [4,12,13]. These emotional struggles are especially strong in patients on hemodialysis, who often face strict schedules, reduced independence, and altered body image from treatment devices [5,10]. In Nepal, patients often face additional burdens. Many must travel long distances to reach dialysis centers, which disrupts their work, income, and family responsibilities [6,14]. Without government coverage for all costs, families face out-of-pocket expenses that make regular treatment difficult [5,6]. Research has found that factors such as low income, poor nutrition, low hemoglobin, lack of education, and advanced CKD stage are strongly linked to worse quality of life (QoL) in CKD patients [5,15,16]. Mental health issues like anxiety and depression significantly reduce QoL and can increase the risk of hospitalization and mortality. Patients without anxiety or depression often have QoL scores similar to healthy individuals [4,17,18].

Despite medical advancements, dialysis technology has had only limited success in improving QoL. Studies show that newer machines and techniques may reduce physical discomfort but do little to improve mental and social well-being [4,19]. In Nepal, where CKD cases are rising, patients often face significant emotional distress and financial strain. Although previous studies [6,14], have identified key factors influencing QoL in patients with CKD including financial hardship, physical symptoms, and emotional burden, there remains a critical research gap in understanding QoL from the patients’ perspectives especially through in-depth qualitative inquiry too. There is a need for mixed-method research to complement quantitative findings with patients’ own lived experiences offering deeper insight and a new dimension to understanding results [14,20]. This approach can help explore more patient-centered and contextually appropriate care strategies. In this regard, this mixed method study aimed to examine the factors associated with QoL of CKD patients & understand their lived experiences.

## Materials and methods

### Study design and setting

This convergent parallel mixed-method study was conducted at the National Kidney Center, a major referral center providing dialysis services in Kathmandu, Nepal from Banasthali and Gaushala where quantitative and qualitative data were collected simultaneously and then analyzed in tandem. A convergent parallel design was chosen because it allowed us to gather both standardized quality of life measures and patients’ personal perspectives at the same time, so the two sets of findings could directly inform each other. This was also the most practical approach within our short study period, helping to reduce the burden on patients during dialysis sessions. By combining numbers with lived experiences, the design gave us a completer and more balanced picture of quality of life than sequential approaches would have. Data collection took place between June 21, 2024, and July 10, 2024. The site was selected due to its high patient turnover and diverse demographics, allowing for a varied sample that represents the hemodialysis patient population coming from Nepal.

### Sample size and sampling procedure

The sample size for the quantitative survey was calculated using Cochran’s formula for estimating a proportion (n = z^2^pq/d^2^), adjusted for a finite population, using a prevalence of 42.9% based on the study done in two national kidney transplantation reference centers of Nepal [21]. The sampling frame for this study consists of all adult patients undergoing maintenance hemodialysis at the National Kidney Center in Kathmandu during the study period. Considering a five percent allowable error, 95% confidence interval & accounting for a 10% non-response error, the sample size was estimated to be 260. The study included adults on hemodialysis for over six months who gave informed consent, were fluent in the assessment language, and could understand and respond to the quality-of-life tool however those who suffering from mentally unstable illnesses that might impact their capacity to give answers were excluded which were verified through medical records alongside observable cognitive impairments. For the qualitative study, a total of 15 participants were purposively selected and approached for in-depth interview (IDI) based on their willingness to be interviewed. Selection also considered clinical stability during the data collection period, the ability to communicate in Nepali and able to share their experiences. Participants were chosen to capture variation in sex, education, socio-economic background, and duration on dialysis for at least six months so as to reflect diverse perspectives. We conducted IDI on quality of life among hemodialysis to provide detailed personal insights, explore complex issues and contextualize quantitative data for a comprehensive understanding. The number of IDIs were determined using the theory of data saturation where no new information emerged. Probes were used to align responses.

### Data collection

Data collection was conducted at the National Kidney Center after receiving approval from the institution’s Research Committee. Face-to-face interviews were carried out during dialysis sessions for both quantitative and qualitative phases of the study. Interviews were collected concurrently to engage and optimize the recruitment process. Validated Nepali versions of the KDQOL – 36 [6,22] and an equity tool were used to evaluate QoL and socio-economic status of hemodialysis patients. For the quantitative information, a semi structured questionnaire (S1 file) consisting three sections were used. First section contained questionnaires on socio demographic characteristics namely sex, marital status, level of education, type of family, household wealth possessions. The second section included questionnaires on behavioral and morbid status namely use of tobacco, use of alcohol, type of diet preferred, time of diagnosis of CKD and felt burden of CKD treatment among respondents. Last section consisted of the Kidney Disease Quality of Life-36 (KDQOL-36) tool, which is a validated, disease-specific questionnaire designed to assess the quality of life in individuals with chronic kidney disease, especially those undergoing dialysis. It includes 36 items that combine general health measures from the SF-12 with kidney-specific domains such as symptom burden, effects of kidney disease, and its impact on daily life. Widely used in clinical and research settings, the KDQOL-36 is valued for its reliability, simplicity, and ability to capture the physical, emotional, and social challenges faced by kidney patients. Each domain of the KDQOL-36 is scored on a 0–100 scale, with higher scores reflecting better perceived quality of life [23]. In this study, we summarized scores using the observed minimum and maximum values, median, and interquartile range (Q3–Q1), given the non-parametric distribution of the data. The KDQOL-36 domains served as the dependent variables, while the variables from the first and second sections were considered independent variables assumed to influence the quality of life among respondents. The average time taken for the interview is about 35 minutes. For qualitative phase, an in-depth interview technique was used to gather detailed and comprehensive information from a small number of participants undergoing hemodialysis. The primary goal was to gain a deeper understanding of the quality of life among these patients by capturing their personal insights, lived experiences, and emotional responses related to kidney disease. The rationale for using in-depth interviews was to provide rich, personal narratives that could help explore complex issues and contextualize quantitative findings, thereby offering a more holistic view of the patients’ quality of life. This qualitative approach allowed for the exploration of subjective dimensions that are often missed in standard surveys or structured questionnaires. A total of fifteen participants were selected for the in-depth interviews. All were patients receiving treatment at the National Kidney Center. Before beginning, the purpose and rules of the interviews were explained to the participants to ensure informed consent and comfort. Each interview session lasted approximately 20 minutes. Semi-structured interviews were guided by five broad questions aligned with KDQOL-36 domains, covering physical health, symptom burden, effects of kidney disease on daily life, mental well-being, and social/family support (e.g., How has your physical health and daily functioning been affected?; What symptoms impact your quality of life the most?; How has kidney disease influenced your ability to perform daily activities, work, or household tasks?; How has kidney disease and dialysis affected your emotional well-being and stress levels?; How do family, friends, or your social environment help you cope, and how has your social life been affected?).We determined the number of interviews based on data saturation, the point when no new themes or insights were emerging, and responses began to repeat what earlier participants had already shared. Saturation was assessed by reviewing transcripts and codes after each set of interviews, and once patterns stabilized, we concluded that additional interviews would not add new information. The interviews were recorded, translated into English, and carefully reviewed to extract key findings. These narratives contributed significantly to understanding the multifaceted experiences of hemodialysis patients.

### Data management and analysis

Quantitative data were entered in Epi Data version 3.1 exported to Statistical Package for Social Science (SPSS) version 22 for analysis (S1 Data). Descriptive statistics were used to summarize the respondents’ demographic profiles, behavioral and morbid status, and quality of life scores. Inferential statistics, specifically the Mann-Whitney U test and Kruskal-Wallis test at 5% level of significance, were applied where appropriate to examine associations between the independent variables and quality of life outcomes.

The audio recordings of in-depth interviews (IDIs) were transcribed in Nepali and then translated to English by first and last author. The transcripts were cross checked for accuracy and language translation consistency from Nepali to English. Prolonged and dedicated involvements of the authors were done throughout the process of transcribing and translating the recordings. The responses were deductively coded into the four domains of the KDQOL. The coding was reviewed again by the authors to understand the emotions and experiences of participants to become familiar with the context. We created data interpretation from respondent’s perspectives that included textual and structural explanations. Since the responses related to symptoms and effects were largely similar, we combined them and incorporated the effect-related responses into the symptom’s domain. Special attention was given to preserving the authenticity of participants’ verbatim responses while systematically aligning the content with the established domains of quality of life. Quotes assigned to each domain were coded using phenomenological approach [24]. The transcripts were returned to the participants for the validation of their responses.

### Ethical consideration

Ethical clearance was obtained from the Institutional Review Committee of Star Hospital (Registration Number: 306/080/081), with additional approval from the National Kidney Center. All participants provided written informed consent, documented by signed consent forms stored securely by the research team. Each consent form was signed by the participant and, when applicable, witnessed by a family member who accompanied the participant during the consent process. Participation was voluntary, with the option to withdraw at any time without explanation. Privacy and confidentiality were strictly maintained, and all participants were treated equally regardless of gender, race, or caste. No one was forced to participate. For in-depth interviews, separate written consent was also obtained for audio recording, and all recordings were anonymized to protect participant identity.

## Findings

This table outlines key characteristics of respondents, including demographic details and health-related factors. The majority of respondents are male (64.2%), with 35.8% female. Most are married or in a domestic relationship (76.5%), while fewer are single (18.5%). In terms of education, 25.4% have no formal education, and 22.7% have completed higher secondary education, with a smaller percentage holding bachelor’s or master’s degrees. Regarding family structure, 42.7% live in single-family units, 35.4% in joint families, and 21.9% in extended families. The majority of respondents (74.2%) report experiencing treatment burden, and a vast majority (95.8%) do not use tobacco, with only a small portion using alcohol (3.1%). Diet-wise, most follow a mixed diet (88.1%), while 11.9% are vegetarian. Regarding kidney disease, most respondents have been diagnosed for over a year, and all were in advanced stage of chronic kidney disease (CKD). In terms of socio-economic status, the respondents are fairly evenly distributed across the five quintiles, with 20.8% in the first quintile and 19.6% in the fifth quintile (Table 1).

**Table 1:** Socio demographic, behavioral & disease related characteristics.

S.N.	Characteristics	Frequency	Percent	S.N.	Characteristics	Frequency	Percent
1	**Sex of the respondents**			6	**Treatment Burden**		
	Male	167	64.2		Yes	193	74.2
	Female	93	35.8		No	67	25.8
2	**Marital status**			7	**Use of Tobacco**		
	Single	48	18.5		Yes	11	4.2
	Married or in a domestic relationship	199	76.5		No	249	95.8
	Divorced or separated	3	1.2	8	**Use of Alcohol**		
	Widowed	10	3.8		Yes	8	3.1
3	**Level of education**				No	252	96.9
	No education	66	25.4	9	**Type of Diet**		
	Able to read and write	26	10		Veg	31	11.9
	Secondary education	47	18.1		Mix	229	88.1
	Higher secondary education	59	22.7	10	**Diagnosed with CKD**		
	Bachelors level	34	13.1		6 months to 1 year	33	12.7
	Master’s degree	28	10.8		1-5year	87	33.5
4	**Type of family**				More than 5 years	140	53.8
	Single	111	42.7				
	Joint	92	35.4				
	Extended	57	21.9				
5	**Socio Economic Status (Quintiles)**						
	First quintile	54	20.8				
	Second quintile	56	21.5				
	Third quintile	46	17.7				
	Fourth quintile	53	20.4				
	Fifth quintile	51	19.6				

The Symptoms/Problem List scores ranged from 2.08 to 100, with a median of 66.67 and an IQR of 31.25, indicating moderate symptom severity and variability in how patients experience symptoms. The Effects of Kidney Disease ranged from 0 to 100, with a median of 59.38 and an IQR of 37.5, reflecting that most patients experience moderate effects, though variability is substantial. The Burden of Kidney Disease ranged from 0 to 75, with a median of 0 and an IQR of 25, suggesting that while most patients report minimal burden, a subset experiences a higher disease burden. SF-12 Physical Composite scores ranged from 15.92 to 58.03, with a median of 32.62 and an IQR of 13.13, indicating moderate physical health and relatively lower variability compared to other domains. Lastly, SF-12 Mental Composite scores ranged from 14.71 to 62.99, with a median of 39.4 and an IQR of 15.09, reflecting moderate mental health with some variability in the central distribution of scores (Table 2).

**Table 2:** Quality of life domain scores.

S.N.	Scale (number of items in scale)	Min	Max	Median	Inter-quartile range (Q3–Q1)
1	Symptoms/problem list	2.08	100	66.67	31.25
2	Effects of kidney disease	0	100	59.38	37.5
3	Burden of kidney disease	0	75	0	25
4	SF-12 Physical composite	15.92	58.03	32.62	13.13
5	SF-12 Mental composite	14.71	62.99	39.4	15.09

Significant differences were found in sex for the Symptom/problem list, Effects of kidney disease, and Burden of kidney disease, with males reporting higher scores, indicating more severe symptoms and disease effects (p < 0.05), while no significant differences were observed in the physical or mental health scores. Marital status showed no significant differences across any domains (p > 0.05). Individuals with formal education reported better outcomes on the Symptoms/problem list and Burden of kidney disease (p < 0.05), but no significant differences were found in physical or mental health scores (p > 0.05). The type of family (single, joint, or extended) did not have a significant effect on any of the domains, except for the SF-12 Mental Composite Score (p = 0.977). Socio-economic status had a strong influence, with individuals in higher quintiles reporting better outcomes across all domains, particularly the Symptoms/problem list and Effects of kidney disease (p < 0.0001), although there was no significant difference for the SF-12 Physical Composite (p = 0.633). The presence of a financial burden due to treatment significantly worsened outcomes in most domains, particularly in symptoms and burden of kidney disease (p < 0.05), with a notable effect on the SF-12 Mental Composite (p < 0.0001). The use of tobacco did not show significant differences in most domains, except for the Burden of kidney disease (p = 0.104). Alcohol consumption was associated with higher burden scores (p = 0.035), but no significant differences were found in the other domains. No significant differences were observed between those preferring a vegetarian or mixed diet (p > 0.05). The length of time diagnosed with CKD did not show significant differences for most domains (p > 0.05). When comparing the subgroups, the medians show the typical score for each group. However, people’s actual scores varied quite a bit around those medians, as seen in the overall interquartile ranges. This means that even if two groups had different medians, their score ranges often overlapped, showing that patients experienced the disease in diverse ways (Table 3).

**Table 3:** Association of independent variables with quality-of-life domains.

S.N.	Characteristics	Symptom/ problem list	Effects of kidney disease	Burden of kidney disease	SF-12 Physical Composite Score	SF-12 Mental Composite Score
**1**	**Sex**					
	Male (Median)	68.75	62.50	6.25	32.68	39.29
	Female (Median)	64.58	53.13	0	32.47	39.72
	Mann-Whitney U	6447	6433	6612.5	7716	7660.5
	Z-value	−2.27	−2.295	−2.142	−0.085	−0.181
	p-value	0.023*	0.022*	0.032*	0.932	0.857
**2**	**Marital Status**					
	Single/Divorced/widower (median)	70.83	59.38	0	32.68	39.7
	Married (median)	66.67	59.38	0	32.56	39.31
	Mann-Whitney U	5483.5	5987.5	5747	6034	5888
	Z-value	−1.141	−0.16	−0.678	−0.069	−0.353
	p-value	0.254	0.873	0.498	0.945	0.724
**3**	**Educational Status**					
	No formal education (Median)	63.54	56.25	0	34.425	36.405
	Have formal education (Median)	68.75	59.38	6.25	32.45	39.835
	Mann-Whitney U	5133.5	5375.5	4914.5	6243	5481.5
	Z-value	−2.405	−1.947	−3.044	−0.301	−1.744
	p-value	0.016*	0.052	0.002*	0.763	0.081
**4**	**Type of family**					
	Single (median)	68.75	59.38	0	33.77	39.8
	Joint (median)	66.67	59.38	0	32.355	39.2
	Extended (median)	70.83	56.25	6.25	30.32	39.26
	Kruskal Walis H Value	0.427	0.933	0.698	1.756	0.047
	degrees of freedom (df)	2	2	2	2	2
	p-value	0.808	0.627	0.705	0.416	0.977
**5**	**Socio economic status**					
	First quintile	56.25	43.75	0	31.16	33.325
	Second quintile	67.71	56.25	0	32.33	41
	Third quintile	65.625	62.5	6.25	33.59	38.215
	Fourth quintile	68.75	59.38	6.25	32.24	39.49
	Fifth quintile	79.17	71.88	18.75	34.34	43.69
	Kruskal Walis H Value	20.701	27.073	19.555	2.561	22.713
	degrees of freedom (df)	4	4	4	4	4
	p-value	<0.0001*	<0.0001*	<0.0001*	0.633	<0.0001*
**6**	**Financial Burden incurred by treatment**					
	Yes (median)	64.58	53.13	0	31.87	36.55
	No (median)	75	71.88	18.75	34.32	43.72
	Mann-Whitney U	4900.5	4085.5	4065.5	5435.5	3560.5
	Z-value	−2.953	−4.492	−4.887	−1.942	−5.478
	p-value	0.003*	<0.0001*	<0.0001*	0.052	<0.0001*
**7**	**Use of Tobacco**					
	Yes (median)	70.83	65.63	12.5	29.73	41.8
	No (median)	66.67	59.38	0	33.02	39.29
	Mann-Whitney U	1327.5	1260.5	1002.5	1142.5	1258.5
	Z-value	−0.172	−0.447	−1.624	−0.93	−0.455
	p-value	0.863	0.655	0.104	0.352	0.649
**8**	**Consumption of Alcohol**					
	Yes (median)	70.83	68.75	21.875	32.945	43.595
	No (median)	66.67	59.38	0	32.55	39.275
	Mann-Whitney U	887	730.5	599	910	639
	Z-value	−0.578	−1.327	−2.109	−0.468	−1.762
	p-value	0.563	0.185	0.035*	0.64	0.078
**9**	**Type of Diet/Food preferred**					
	Veg (median)	66.67	46.88	0	29.73	38.01
	Mix (median)	66.67	59.38	6.25	33.02	39.49
	Mann-Whitney U	3526	2991.5	2866	2857	3309.5
	Z-value	−0.06	−1.422	−1.878	−1.762	−0.611
	p-value	0.952	0.155	0.06	0.078	0.541
**10**	**Diagnosed with Chronic Kidney Disease**					
	6 months – 1 year (median)	77.08	62.5	6.25	32.77	39.26
	1-5 years (median)	68.75	59.38	0	35.04	39.01
	More than 5 years (median)	66.67	59.38	0	31.495	39.97
	Kruskal Walis H Value	3.114	0.259	1.278	2.428	0.858
	degrees of freedom (df)	2	2	2	2	2
	p-value	0.211	0.878	0.528	0.297	0.651

*Statistically significant at 5% level.

### Qualitative findings on physical health-related quality of life in hemodialysis patients

Patients undergoing hemodialysis frequently reported persistent fatigue, anorexia, and significant weight loss, all of which severely impacted their daily lives. Many struggled with exhaustion, making even simple tasks feel overwhelming. Routine activities such as lifting small objects or walking short distances required frequent rest breaks, and some patients had to rely on family members for assistance. Muscle weakness and reduced stamina further restricted their mobility, often leading to frustration and a sense of dependency.

A common experience among patients was drastic weight fluctuations, contributing to their overall physical decline. One participant shared, *“I didn’t eat much, and I couldn’t sleep well at night due to chest congestion. My weight dropped from 76 kg to 39 kg. Eventually, I started eating a little and began to recover. Now, I weigh around 75 kg.”* (IDI:9). Another echoed a similar experience, *“Initially, I weighed 101 kilos, then dropped to 43 kilos. Now, I weigh 61 kilos.”* (IDI:6). Beyond weight loss, patients reported a significant decrease in endurance and strength, affecting their ability to engage in daily activities. One noted, *“I don’t have the same enthusiasm as before, and it is difficult to work. My endurance has decreased, and I find it harder to walk or move around for dialysis.”* (IDI:12). Another explained, *“My strength has gradually decreased. I used to be strong, but now I feel weaker and experience more pain and difficulty.”* (IDI:13).

Many patients reported difficulties with mobility, stating that even simple movements had become challenging. Some explained that missing a single dialysis session made breathing difficult, while others experienced a significant decline in their ability to stand or walk for extended periods. One patient shared, *“It’s very difficult. For example, I can’t walk now. If I miss a dialysis session, breathing becomes hard. When my blood level decreases, my energy drops, and I can’t perform the activities I used to. Now, I can only do simple tasks like sitting in a shop and handing out small items. The doctor advises not to lift more than two kilos. It’s very tough.”* (IDI:7). Another added, *“If I stand for a while, my legs get weak.”* (IDI:10).

Patients also described a drastic decline in their endurance compared to before starting hemodialysis. One reflected, *“There is a big difference between working for 24 hours and working for 4 hours; it impacts blood volume immensely. You can’t eat certain foods, you feel weak, and the entire lifestyle is affected.”* (IDI:3). Another noted, *“Now, my memory is also decreasing. Endurance is tied to a person’s nature, understanding, and practical behavior. It’s not like it used to be when I was normal. That’s not possible anymore.”* (IDI:7).

Pain and discomfort were commonly reported, with many experiencing persistent muscle cramps, joint pain, and general weakness. These symptoms forced patients to modify their daily routines and incorporate frequent rest breaks. One participant described, *“Once you get sick, your physical strength naturally decreases. My body has become weaker, and after starting dialysis, my bones have weakened, and my skin itches.”* (IDI:1). Another added, *“Before starting dialysis, I was able to do more, but now I can hardly stand. My arms and legs feel weak, my bones feel eroded, and many other problems have arisen. There isn’t anything that hasn’t been affected.”* (IDI:2).

In addition to these physical challenges, patients reported a decline in social interaction and a reduction in their ability to engage in social or work-related activities. One participant shared, *“It has changed a lot. Now, no one is around except my brother, sister, and close relatives. I don’t know where my friends are. Only my husband is there for me.”* (IDI:4). Another described how their daily routine was altered: *“It’s hard now. I can’t go out and work. If I try to work, I miss my dialysis. If I miss it, I could die. I can’t do household chores as I used to. Earlier, I could do everything, like washing clothes, buying groceries, cooking.”* (IDI:6).

Despite these limitations, some patients adopted strategies to maintain independence. While many modified their daily routines to conserve energy, others engaged in light physical activities such as stretching or slow walking to manage stiffness and improve circulation. However, the persistent burden of fatigue, mobility challenges, and physical discomfort continued to hinder their ability to carry out basic daily tasks, with many relying on caregivers or external support.

### Qualitative findings on mental health-related quality of life in hemodialysis patients

The unpredictability of hemodialysis treatment and frequent hospital visits contributed to persistent anxiety and depressive symptoms. Many patients expressed feelings of hopelessness, emotional distress, and anxiety about their condition, financial burden, and future uncertainties. The study findings indicate that patients with higher socio-economic status had significantly better mental health scores (**p < 0.0001**), suggesting that financial stability plays a crucial role in mental well-being. One participant described their persistent anxiety: *“I feel worried and in pain. I get anxious about what I need to do.”* (IDI:12). Another shared how dialysis initially led to a mental health decline: *“It completely affects you; you feel like everything is over, like there’s nothing left. Gradually, as you continue dialysis, you develop hope that you can move forward with a transplant. Initially, it feels devastating with depression and anxiety, and your dreams are shattered.”* (IDI:3). A similar sentiment was echoed by another participant: *“I used to have a lot of anxiety, thinking the world was ending. It was very difficult, and I still experience it, but now I’ve come to terms with it.”* (IDI:13).

The emotional toll of kidney disease was also evident in patients’ responses, with many expressing stress, anxiety, and frustration. One participant explained their initial worry: *“I don’t know what I was worried about, but I was worried (laughs). I thought I would always have to do dialysis. At first, I didn’t know much, and it was very hard. I thought I would always have to live like this, and just when I was at the age to study, I became ill, which caused me a lot of worry.”* (IDI:4). Another patient voiced concern over financial issues: *“I worry about not having enough money for treatment. This illness makes me worry about what to eat and how to manage everything.”* (IDI:9). A patient further reflected on the broader impact of illness, saying, *“Life changes after getting sick. It’s not like being healthy. One has to go through different stress and problems.”* (IDI:11).

Despite these challenges, some patients reported a shift toward emotional stability over time. One individual explained, *“Initially, you have to accept it. Once you accept it, you understand how to move forward and how to reduce complications by focusing on diet and other aspects.”* (IDI:3). Another added, *“Initially, people experience depression and anxiety. After overcoming the initial phase, stability comes. It’s still ongoing like that, and now it’s somewhat stable.”* (IDI:3). Some patients even described a sense of relief after dialysis: *“Dialysis makes me feel lighter, and my mood doesn’t get as bad as it used to.”* (IDI:9).

Despite the mental and emotional burden, many patients demonstrated resilience through adaptive coping strategies, including seeking social support, engaging in religious practices, and maintaining a positive outlook. Some participants reported a shift in mindset that helped them endure the treatment. One individual shared, *“I used to worry about my child becoming an orphan, but I realized that I need to stay strong for their sake.”* (IDI:2). Another expressed a similar sentiment: *“I have to accept that I need to live. Whatever happens, I have to endure it and move forward with energy.”* (IDI:7).

Family and social support played a crucial role in helping patients manage their emotional distress. Many participants emphasized how the presence of supportive family members alleviated their fears and stress. One participant reflected, *“Mentally, I haven’t experienced much pressure, maybe because my family supports me well. Someone is always there to console me, so I don’t feel much mental stress. I was very scared about what dialysis would be like. My family didn’t give me much time to think about it, except at night when I couldn’t sleep and would cry, thinking about my child being left alone. But after my family decided on a transplant, I felt better. It didn’t work out, but I felt less tense.”* (IDI:2). Another patient emphasized the importance of emotional support from loved ones: *“The biggest support comes from family and friends. Their support and consolation are what keep us going. Having people who stand by you during difficult times helps a lot.”* (IDI:3).

Several participants highlighted the constant presence of their loved ones during difficult moments: *“For about 1 to 1.5 months, my husband never left me alone, even when I went to the bathroom or washed my face. I had a sister who did dialysis with me, and she tried to comfort me a lot. The doctors also advised me a lot, saying I shouldn’t take certain medications and should control myself. I received a lot of support from them. My sisters also advised me to control myself, and later I understood. Everyone supported me, and after thinking about it a lot, I felt like crying. But with the support of my family, I managed.”* (IDI:4).

In addition to emotional and family support, some patients actively used distraction techniques to manage stress and maintain a sense of normalcy. Engaging in light household tasks, watching entertainment, and keeping occupied with hobbies were commonly reported coping mechanisms. One participant shared, *“I try to distract myself by watching comedy on my phone and doing household chores. Sometimes I cook something I crave.”* (IDI:10). Another explained, *“I distract myself by using my phone, watching educational videos, and other things to keep myself occupied.”* (IDI:11). Others focused on light physical activities to shift their attention away from their illness: *“I try not to think too much about it and keep myself busy with light household tasks like dusting and organizing clothes to distract myself.”* (IDI:12).

Despite the significant psychological and emotional challenges faced by hemodialysis patients, the presence of social support, adaptive coping mechanisms, and financial stability played a crucial role in maintaining psychological resilience. While many patients initially struggled with anxiety and depression, those who actively engaged in positive coping strategies, received strong family support, and maintained a hopeful outlook were better able to manage their emotional distress.

### Qualitative findings on the symptoms domain in hemodialysis patients

Patients undergoing hemodialysis experience a range of physical symptoms, including persistent fatigue, pain, discomfort, sleep disturbances, and limitations in daily activities. These symptoms contribute significantly to their overall symptom burden, with some reporting severe impacts on their quality of life. The Symptoms/Problem List domain in this study ranged from 2.08 to 100, with a mean of 65.60 ± 21.21, indicating moderate to severe symptom burden with high variability among patients.

Many participants shared their struggles with chronic pain, muscle weakness, and severe fatigue, which hindered their ability to perform everyday tasks. One patient described the discomfort: *“The skin itches, there’s dizziness, and if too much fluid is removed, it can cause low pressure and weakness. All of these happen.”* (IDI:1). Another participant expressed similar discomfort, saying, *“I feel nauseous, have body aches, itchy skin, dry skin, and loss of appetite.”* (IDI:15).

In addition to physical discomfort, some patients also faced complications that exacerbated their symptoms. One patient explained: *“I had severe itching due to high phosphorus levels. If not controlled, protein deficiency can cause swelling. Other symptoms include bone pain, itching, and mental distress. I have difficulty sleeping, lack of appetite, restlessness, and leg problems.”* (IDI:8). Another patient shared, *“Sometimes my blood pressure drops, and I feel dizzy. Occasionally, my hands and feet hurt.”* (IDI:9).

Managing these symptoms is crucial for improving patients’ well-being. Many relied on medications or non-medical coping strategies to alleviate discomfort. One patient described using physical remedies: *“By rubbing the itchy skin against the wall, stretching and pressing on cramps, and getting massages.”* (IDI:7). Another patient said they managed their symptoms through medication: *“I take medication for itching and calcium supplements. I also take protein supplements and manage frequent urinary infections.”* (IDI:5).

Despite the persistent nature of these symptoms, patients developed coping mechanisms to manage their physical discomfort. Some patients adjusted their daily routines, incorporated rest periods, and sought support from family or fellow dialysis patients. One patient shared, *“I used to feel lonely and sad, but now I have friends and support from other dialysis patients. I have built up my morale and realized that life goes on.”* (IDI:5). Another emphasized resilience, stating, *“I will live as long as my time allows. I’ve accepted it. As long as I live, I will keep going. I don’t let negative thoughts come to my mind.”* (IDI:10).

Family support played a key role in helping many patients cope with the symptoms of hemodialysis. One participant described, *“Mentally, I haven’t experienced much pressure, maybe because my family supports me well. Someone is always there to console me, so I don’t feel much mental stress. My family didn’t give me much time to think about it, except at night when I couldn’t sleep and would cry, thinking about my child being left alone. But after my family decided on a transplant, I felt better. It didn’t work out, but I felt less tense.”* (IDI:2).

Moreover, financial stability also emerged as an important factor in managing symptoms. Participants with better socio-economic status reported significantly lower symptom severity scores, suggesting that financial security may alleviate some of the burden caused by the illness.

In conclusion, the symptoms experienced by patients undergoing hemodialysis are complex and multifaceted, impacting both their physical health and emotional well-being. However, adaptive coping mechanisms, family support, and symptom management strategies are crucial in helping patients cope with their daily challenges. Furthermore, socio-economic factors, including financial stability, play a vital role in reducing symptom severity and improving overall quality of life.

### Qualitative findings on the Burden/effect domain in hemodialysis patients

Many patients reported feeling overwhelmed by the relentless demands of treatment, frequent medical visits, and financial constraints, all of which significantly impact their mental, physical, and social well-being.

The emotional and psychological burden was a dominant theme, with patients expressing feelings of helplessness, exhaustion, and stress due to the ongoing nature of their illness. The association between financial burden and higher disease burden scores (p < 0.0001) highlights how economic challenges exacerbate distress. Many patients viewed the future with uncertainty and hopelessness, as reflected in statements like *“We have to continue dialysis to live. How can we feel about the future? We sick people have a miserable life. It will always be bad. We have to continue dialysis until we die”* (IDI:4). Others expressed anxiety about dialysis-related complications: *“I’ve seen people die from dialysis-related complications. Seeing others die like that makes me think it might happen to me too. It’s something I have to face and try to understand.”* (IDI:6). The unpredictability of their condition led to a sense of resignation, with another patient stating, *“The future seems bleak. We don’t see any aim or hope”* (IDI:7).

Dependency on treatment and loss of independence added to the overall burden. The necessity of dialysis dictated patients’ daily schedules, limiting their ability to engage in personal or professional aspirations. One participant shared, *“I can’t think about doing things like playing football or singing. My focus is on sustaining life, creating schedules, and thinking about how to survive. I had to return from London, where I was studying HRM. It was a significant loss, but I had to continue my treatment”* (IDI:3). Another participant emphasized how dialysis had become an unavoidable part of life: *“This is my future—continuous dialysis. I can’t do anything else. It’s necessary to keep doing dialysis, and I feel like this is normal now. There’s no point in worrying about the future; whatever happens, happens”* (IDI:12).

A significant financial burden further intensified patients’ struggles, with economic constraints playing a major role in shaping their experience. Higher socioeconomic status was associated with significantly lower disease burden scores (p < 0.0001), suggesting that financial stability eases some of the challenges of long-term dialysis. Those facing financial difficulties expressed constant worry about affording treatment and daily necessities. One participant admitted, *“I worry about not having enough money for treatment. This illness makes me worry about what to eat and how to manage everything”* (IDI:9).

Despite these difficulties, coping strategies and adaptation mechanisms emerged, with some patients finding solace in family support, positive thinking, and acceptance. Family support was frequently cited as a crucial factor in reducing emotional distress. One participant shared, *“Mentally, I haven’t experienced much pressure, maybe because my family supports me well. Someone is always there to console me, so I don’t feel much mental stress”* (IDI:2). Another emphasized the role of loved ones in sustaining morale: *“The biggest support comes from family and friends. Their support and consolation are what keep us going. Having people who stand by your during difficult times helps a lot”* (IDI:3).

Positive thinking was another key coping mechanism, with patients striving to remain hopeful despite the uncertainties of their condition. One participant said, *“I used to worry about my child becoming an orphan, but I realized that I need to stay strong for their sake”* (IDI:2), while another affirmed, *“I have to accept that I need to live. Whatever happens, I have to endure it and move forward with energy”* (IDI:7). Some patients attempted to normalize their condition, accepting their circumstances with resilience. One patient reflected, *“I used to have a lot of anxiety, thinking the world was ending. It was very difficult, and I still experience it, but now I’ve come to terms with it”* (IDI:13). Another acknowledged their situation with pragmatism: *“I believe that everyone has to die one day, whether they have a disease or not”* (IDI:2).

## Discussions

In this study, the Symptoms/Problem List was the best-performing (median = 66.67 IQR = 31.25), indicating that many patients were able to manage or adapt to physical discomforts such as itching, fatigue, and muscle pain. The relatively higher median score for symptoms shows that while physical symptoms are prevalent, many patients have developed personal, family-based, or behavioral coping strategies to manage them. Qualitative accounts converged with these quantitative findings, as participants described concrete coping strategies:, *“I press my muscles when they cramp. I take protein and calcium and stretch when I feel pain”* (IDI:7). Another explained, *“After dialysis, I feel lighter”* (IDI:9), suggesting adaptation and symptom relief post-treatment. In Nepal, two studies did not assess this domain specifically through KDQOL-36, but reported moderate scores in physical domains, supporting that physical discomforts are less distressing than emotional and financial burdens [6,21]. Another study found that the symptoms domain was also the best-performing, attributed to routine symptom control and high dialysis adherence [10]. A multi country study also found symptom scores higher than in this study, but the rank of the symptom domain remained best-performing globally [17]. While symptom control remains a challenge, it is consistently the most manageable domain across both local and global contexts likely because symptoms are predictable, medicalized, and often short-term compared to broader burdens. At the same time, a divergence is evident. While the symptom scores were the highest overall, the personal stories remind us that living with these discomforts is far from effortless. Muscle cramps, fatigue, and the need for constant self-care show that symptoms remain an everyday struggle. This suggests that the numbers alone may not fully capture the ongoing effort patients put into managing their bodies and maintaining a sense of normalcy.

In contrast, the Burden of Kidney Disease scored extremely low (median = 0, IQR = 25), highlighting how dialysis patients face chronic emotional stress, dependence, loss of autonomy, and severe financial pressure. Multiple patients described the relentlessness of treatment: *“This is my future now – just dialysis. I can’t think beyond survival”* (IDI:3),

*“We sick people have a miserable life. It will always be bad”* (IDI:4). The quantitative and qualitative findings converge, i.e., low median scores mirror patients’ experiences of stress, dependence, and loss of autonomy. A divergence is also apparent, as personal accounts reveal ongoing despair and the daily effort required to manage chronic illness, showing that numbers alone cannot fully capture the depth of distress. The strands complement each other by adding context, i.e., narratives explain why the burden is so heavy, highlighting relentless treatment, financial pressures, and limited emotional support, and pointing to structural gaps in mental health services, social insurance, and rehabilitation that intensify struggles in Nepal. Similar patterns have been observed in other studies too. In one study, patients experiencing high out-of-pocket expenses, transportation challenges, and unemployment reported low psychological and social quality of life (QoL), highlighting the impact of burden-related distress, even though it was not assessed as a separate domain [6]. Another study also reported lower psychological and social domain scores among dialysis patients than transplant recipients, emphasizing emotional burnout due to lifelong treatment dependency [21]. An Indian study divulged that burden of disease had the lowest median score (median = 0), showing that even in a relatively better-resourced setting, emotional burden was hardest to address [10]. A multi country study also revealed global burden scores were much higher but still lower than symptom scores, confirming that burden is a universally low domain [17]. However, the discrepancy in absolute score highlights Nepal’s structural limitations compromised mental health support, social insurance, or under resourced rehabilitation services intensifies distress. These comparisons confirm that while burden is globally the most impacted domain, it is disproportionately severe in Nepal owing to weaker financial protections and poor integrated care particularly for chronically ill.

In our study, sex was significantly associated with the Symptom, Effects, and Burden domains of QoL, with males reporting higher median scores indicating that men perceived less impact from kidney disease. This aligns with findings from Nepal where women consistently reported lower physical and psychological QoL, likely due to limited financial autonomy, higher caregiving responsibilities, and social isolation [6,21]. Similarly, a study in India observed that dialysis depended female patients had poorer scores in mental and burden domains, attributed to traditional gender roles and dependence on others for healthcare access [10]. Globally, a multi country study also found that males had slightly better QoL scores across domains, including symptoms and burden, although the gender gap was less pronounced suggesting that universal healthcare and stronger psychosocial support systems in high-income countries may help reduce sex-based disparities [17]. These findings stress the need for gender-responsive policies in dialysis care, including mental health counseling, social support networks, and interventions that fosters women’s independence and engagement in their treatment.

In our study, socio-economic status (SES) was significantly associated with all QoL domains except physical health, with patients in the highest quintile reporting notably higher scores compared to those in the lowest quintile (p < 0.0001). This trend aligns with a study done in Nepal, which found that low-income patients in Nepal experienced lower QoL due to transportation challenges, malnutrition, and financial strain [6]. Similarly, another study reported higher QoL scores among patients with better economic status, especially in physical and psychological domains [21]. In India, a study also observed that patients from higher SES backgrounds had better scores, attributing this to improved access to healthcare, medication, and diet [10]. A systematic review showed that globally, the vast majority of dialysis patients in low-income countries lacked access to treatment up to 96% in some settings resulting in millions of preventable deaths, while patients in wealthier nations had significantly better access due to stronger health systems and economic capacity [25]. These findings strongly suggest that SES is a cross-cutting determinant of QoL, and call for policy-level interventions exploring on innovative and cost-effective ways of delivering dialysis and post dialysis care including psychosocial support to improve QoL outcomes for economically disadvantaged dialysis patients. A mixed method study further emphasized that patients often experience internal suffering that remains masked under external compliance [12].

Our study divulged that several patients, especially women, voiced feelings of social isolation, with one saying, *“Now, no one is around except my husband”* (IDI:4), reflecting the erosion of social networks. This aligns with the quantitative findings: the SF-12 Mental Composite score was relatively low (median = 39.4, IQR = 15.09), indicating compromised emotional and social functioning, while the Effects of Kidney Disease domain had a moderate score (median = 59.38, IQR = 37.5), suggesting that CKD impacts daily activities and social engagement. This convergence between numbers and narratives highlights how social isolation and emotional strain are tangible aspects of patients lived experience. Similar pattern was also noted in a study in Nepal, where rural patients had reduced community engagement due to treatment logistics and stigma [6]. A divergence also emerges. While the Effects of Kidney Disease scores suggest moderate functional impact, the qualitative accounts reveal deeper experiences of stigma, loneliness, and ongoing emotional burden that numerical scores alone cannot fully capture. At the same time, some participants demonstrated resilience. One explained, “*I have to accept it and move forward with energy*” (IDI:7), showing adaptive coping strategies. This complementarity, between patients’ stories and quantitative trends, illustrates why some individuals maintain psychological well-being despite social and emotional challenges, often supported by family or community networks, as also observed in multicounty study [17]. Overall, the IDIs highlighted the multifaceted impact of CKD beyond clinical symptoms, with emotional, social, and economic dimensions often compounding patients’ suffering, calling for more holistic, patient-centered care approaches in Nepal’s dialysis programs.

The strengths of this study are study employed a convergent parallel mixed-method design, which enabled the integration of both quantitative data and rich qualitative insights. This combination enhanced the depth and contextual validity of the findings. The study used a validated tool which ensured culturally appropriate and reliable measurement of QoL among hemodialysis patients. The study included participants from various socio-economic backgrounds and both urban and semi-urban settings, enhancing generalizability within the Nepalese context. The qualitative component captured detailed personal experiences, revealing deeper emotional, physical, and financial burdens that are often missed in quantitative assessments alone thereby could potentially intervene patient centered care approach. Ethical protocols, including informed consent and respondent validation of qualitative transcripts, upheld participant autonomy and credibility of data interpretation. However, some limitations should be considered. Even though centers are large and serve many patients, they are located in urban areas therefore, the results might not fully reflect the situation of patients living in remote or rural areas of Nepal. Also, because the study was cross-sectional (done at one point in time), we couldn’t track changes in quality of life over time. Some responses may have been influenced by recall bias or by how comfortable patients felt sharing sensitive information. In addition, as the KDQOL-36 is a self-reported tool, it may not fully capture objective health status, especially among patients with limited literacy. Even with a validated Nepali version, this could introduce some measurement bias. Lastly, we did not explore in depth how dialysis quality, duration, or other health conditions might affect quality of life.

## Conclusion

This mixed-method study highlights that while physical symptoms among hemodialysis patients in Nepal can often be managed through treatment adherence and coping strategies, the burden of disease; particularly in terms of emotional distress, financial hardship, and loss of independence remains the most weakening and under-addressed aspect of quality of life. The findings are consistent with both regional and global studies, yet Nepal displays more extreme lows in burden domain scores, primarily due to limited mental health services, financial protections, and decentralized care options. These results emphasize the need for an integrated, patient-centered care model that addresses the multifaceted needs of dialysis patients, shifting the focus from mere survival to holistic, dignified living.

## Recommendations

The study findings highlight the need for a multi-faceted approach to improve quality of life for dialysis patients in Nepal. While symptom management appears relatively effective, significant challenges remain in addressing the severe emotional and financial burdens, particularly for women and low-income patients. Key recommendations include enhancing symptom management through patient education and palliative care integration, reducing treatment burdens via mental health support and financial assistance programs, and addressing gender disparities through targeted counseling and empowerment initiatives. Accessibility should be improved through subsidized treatment options and nutritional support, particularly for disadvantaged groups. Future efforts should focus on longitudinal research including rural populations and policy reforms such as multidisciplinary care models and a national dialysis registry. These comprehensive interventions would address both the medical and socioeconomic dimensions of kidney disease, ultimately leading to better patient outcomes and quality of life. The findings underscore that while physical symptoms can be managed, the psychological and financial impacts of chronic dialysis require urgent, systemic solutions tailored to Nepal’s healthcare context.

## Supporting information

S1 File Survey questionnaire. (DOCX)

S1 Data (The dataset was originally generated and analyzed in SPSS (.sav format). *For submission purposes, the dataset has been converted to Microsoft Excel (.xlsx) format accompanied by a complete SPSS codebook (variable names, labels, measurement levels, and value labels). The conversion preserves all variables and coding schemes*).
(XLSX)
