# Supplementary material for: Quality of life among hemodialysis patients in a referral center in Kathmandu: A mixed method study
Source: PLoS One. 2025 Nov 3;20(11):e0335990. doi: 10.1371/journal.pone.0335990 (PMC12582446; doi:10.1371/journal.pone.0335990)
Supplement: S1 File — (DOCX) [file pone.0335990.s001.docx]

# Quality of Life Among Hemodialysis Patients in a Referral Center in Kathmandu: A Mixed Method Study

## Section 1: Demographic Information (जनसांख्यिक जानकारी / Demographic Information)

| # | प्रश्न (Question) | उत्तर विकल्प (Response Options) | Tick Box |
| --- | --- | --- | --- |
| 1 | उमेर (Age) | [……………….in completed years] |  |
| 2 | लिङ्ग (Gender) | पुरुष (Male) / महिला (Female) / अन्य (Other) | ☐ ☐ ☐ |
| 3 | परिवारको प्रकार (Family Type) | एकल (Nuclear) / संयुक्त (Joint) / बढि (Extended) | ☐ ☐ ☐ |
| 4 | जात (Ethnicity) | बाहुन/छेदी (Brahmin/Chhetri) / जनजाति (Janajati) / मधेशी (Madhesi) / दलित (Dalit) / अन्य (Other) | ☐ ☐ ☐ ☐ ☐ |
| 5 | शिक्षा स्तर (Education Level) | अशिक्षित (Illiterate) / साधारण लेखपढ (Able to read & write) / मुख्य शिक्षा (Primary 1–8) / माध्यमिक (Secondary 9–10) / उच्च माध्यमिक (Higher Secondary 11–12) / स्नातक (Bachelor) / स्नातकोत्तर ( Master) | ☐ ☐ ☐ ☐ ☐ |
| 6 | वैवाहिक स्थिति (Marital Status) | एकल (Single) / विवाहिता (Married) / साझेदार (Living; partnering) / विधवा (Widowed)/बिछोड (Divorced) | ☐ ☐ ☐ ☐ |

## Socioeconomic Information (सामाजिक–आर्थिक जानकारी / Socioeconomic Information)

| # | प्रश्न (Question) | उत्तर विकल्प (Response Options) | Tick Box |
| --- | --- | --- | --- |
| 1 | घरमा टेलिभिजन छ? (Do you have a television at home?) | छ (Yes) / छैन (No) | ☐ ☐ |
| 2 | घरमा कपबोर्ड छ? (Do you have a cupboard at home?) | छ (Yes) / छैन (No) | ☐ ☐ |
| 3 | घरमा मेज छ? (Do you have a table at home?) | छ (Yes) / छैन (No) | ☐ ☐ |
| 4 | घरमा पंखा छ? (Do you have a fan at home?) | छ (Yes) / छैन (No) | ☐ ☐ |
| 5 | बसोबासको मुख्य फर्श (Main flooring of residence) | माटो/रेत (Mud/Sand) / अन्य (Other) | ☐ ☐ |
| 6 | बाहिरी दिवारको मुख्य सामग्री (Main material of outer wall) | फसेमेन्ट (Cement) / अन्य (Other) | ☐ ☐ |
| 7 | छानाको मुख्य सामग्री (Main roof material) | फसेमेन्ट (Cement) / अन्य (Other) | ☐ ☐ |
| 8 | खाना पकाउने मुख्य ईन्धन (Main cooking fuel) | LPG / काठ (Wood) / अन्य (Other) | ☐ ☐ ☐ |
| 9 | आयको स्रोत (Source of income) | कृषि (Agriculture) / सरकारी नौकरी (Government job) / निजी (Private) / व्यवसाय (Business) / विदेशी रोजगार (Foreign employment) / अन्य (Other, specify) | ☐ ☐ ☐ ☐ ☐ ☐ |

## Section 2: Health Information (रोगसँग सम्बन्धी / Disease Related)

| # | प्रश्न (Question) | उत्तर विकल्प (Response Options) | Tick Box |
| --- | --- | --- | --- |
| 1 | CKD भएको अवधि (Duration of CKD) | ६ महिना–१ वर्ष (6 months–1 year) / १–५ वर्ष (1–5 years) / ५ वर्ष भन्दा बढी (More than 5 years) | ☐ ☐ ☐ |
| 2 | हेमोडायलिसिस अवधि (Duration of Hemodialysis) | ६ महिना–१ वर्ष (6 months–1 year) / १–५ वर्ष (1–5 years) / ५ वर्ष भन्दा बढी (More than 5 years) | ☐ ☐ ☐ |
| 3 | Comorbidities (सामान्य रोगहरू) | उच्च रक्तचाप (Hypertension) / मधुमेह (Diabetes) / अन्य (Other) | ☐ ☐ ☐ |

##

## Lifestyle (जीवनशैली)/Treatment Burden (उपचारको लागत बोझ)

| # | प्रश्न (Question) | उत्तर विकल्प (Response Options) | Tick Box |
| --- | --- | --- | --- |
| 1 | चुरोट / तम्बाकू प्रयोग गर्नुहुन्छ? (Do you smoke or use tobacco products?) | हो (Yes) / होइन (No) | ☐ ☐ |
| 2 | मदिरा सेवन गर्नुहुन्छ? (Do you consume alcohol?) | हो (Yes) / होइन (No) | ☐ ☐ |
| 3 | आहार (Diet) | शाकाहारी (Vegetarian) / मांसाहारी (Non-vegetarian/Mixed) | ☐ ☐ |
| 4 | उपचारको लागतबोझ (cost of burden) | छ (Yes) / छैन(No) | ☐ ☐ |

##

## Section 3: मृगौला रोग र जीवनस्तर (KDQOL-36) प्रश्नावली / Kidney Disease and Quality of Life (KDQOL-36) Questionnaire

## खण्ड १: सामान्य स्वास्थ्य / Section 1: General Health

**१. सामान्यतया, तपाईं आफ्नो स्वास्थ्यलाई कस्तो भन्नुहुन्छ?***1. In general, would you say your health is:*- उत्कृष्ट / धेरै राम्रो / राम्रो / सामान्य / कमजोर / Excellent / Very good / Good / Fair / Poor

**२. तलका क्रियाकलापहरू गर्न तपाईंको स्वास्थ्यले रोक्छ?***2. Does your health limit you in these activities?*- मध्यम क्रियाकलाप (टेबल सार्नु, भ्याकुम प्रयोग गर्नु, बौलिङ, गोल्फ खेल्नु) / Moderate activities (moving a table, using a vacuum, bowling, playing golf)

- - एकदमै रोक्छ / अलिकति रोक्छ / बिलकुलै रोक्दैन / Yes, limited a lot / Yes, limited a little / No, not limited at all

**३. धेरै तल्ला चढ्नु:***3. Climbing several flights of stairs:*- एकदमै रोक्छ / अलिकति रोक्छ / बिलकुलै रोक्दैन / Yes, limited a lot / Yes, limited a little / No, not limited at all

## खण्ड २: शारीरिक स्वास्थ्य (विगत ४ हप्ताभित्र) / Section 2: Physical Health (Past 4 Weeks)

**४. तपाईंले गर्न चाहेजस्तो भन्दा कम गर्नुभयो?***4. Did you accomplish less than you would like?*- हो / होइन / Yes / No

**५. तपाईंले गर्ने काम वा गतिविधिमा सीमित हुनुभयो?***5. Were you limited in the kind of work or activities?*- हो / होइन / Yes / No

## खण्ड ३: भावनात्मक समस्या (विगत ४ हप्ताभित्र) / Section 3: Emotional Problems (Past 4 Weeks)

**६. तपाईंले गर्न चाहेजस्तो भन्दा कम गर्नुभयो?***6. Did you accomplish less than you would like?*- हो / होइन / Yes / No

**७. तपाईंले काम वा गतिविधि सामान्य जस्तो सावधानीपूर्वक गर्नुभएन?***7. Did you not do work or activities as carefully as usual?*- हो / होइन / Yes / No

## खण्ड ४: दुखाइ (विगत ४ हप्ताभित्र) / Section 4: Pain (Past 4 Weeks)

**८. तपाईंको दुखाइले तपाईंको सामान्य काममा कत्तिको असर गर्‍यो? (घर भित्र र बाहिर)***8. How much did pain interfere with your normal work (at home and outside)?*- बिल्कुलै छैन / अलिकति / मध्यम / धेरै / अत्यन्तै / Not at all / A little bit / Moderately / Quite a bit / Extremely

## खण्ड ५: भावनाहरू (विगत ४ हप्ताभित्र) / Section 5: Emotions (Past 4 Weeks)

**९. तपाईं शान्त र शान्त महसुस गर्नुभयो?***9. Have you felt calm and peaceful?*- सधैं / धेरैजसो / प्रायः / कहिलेकाहीँ / अलिकति / कहिल्यै होइन / All of the time / Most of the time / A good bit of the time / Some of the time / A little of the time / None of the time

**१०. तपाईंमा धेरै उर्जा थियो?***10. Did you have a lot of energy?*- सधैं / धेरैजसो / प्रायः / कहिलेकाहीँ / अलिकति / कहिल्यै होइन / All of the time / Most of the time / A good bit of the time / Some of the time / A little of the time / None of the time

**११. तपाईं निराश र उदास महसुस गर्नुभयो?***11. Have you felt downhearted and blue?*- सधैं / धेरैजसो / प्रायः / कहिलेकाहीँ / अलिकति / कहिल्यै होइन / All of the time / Most of the time / A good bit of the time / Some of the time / A little of the time / None of the time

**१२. शारीरिक वा मानसिक समस्याले तपाईंको सामाजिक गतिविधिमा असर गर्‍यो?***12. Did problems interfere with your social activities?*- सधैं / धेरैजसो / प्रायः / कहिलेकाहीँ / अलिकति / कहिल्यै होइन / All of the time / Most of the time / A good bit of the time / Some of the time / A little of the time / None of the time

## खण्ड ६: मृगौला रोगको असर / Section 6: Burden of Kidney Disease

**१३. मेरो मृगौला रोगले मेरो जीवनमा धेरै असर गर्छ।***13. My kidney disease interferes too much with my life.*- पूर्ण सत्य / प्रायःसत्य / थाहा छैन / प्रायः गलत / पूर्ण गलत / Definitely true / Mostly true / Don't know / Mostly false / Definitely false

**१४. मेरो धेरै समय मृगौला रोगसँग जुध्नमै जान्छ।***14. Too much of my time is spent dealing with my kidney disease.*- पूर्ण सत्य / प्रायःसत्य / थाहा छैन / प्रायः गलत / पूर्ण गलत / Definitely true / Mostly true / Don't know / Mostly false / Definitely false

**१५. म मेरो मृगौला रोगसँग जुध्दा निराश हुन्छु।***15. I feel frustrated dealing with my kidney disease.*- पूर्ण सत्य / प्रायःसत्य / थाहा छैन / प्रायः गलत / पूर्ण गलत / Definitely true / Mostly true / Don't know / Mostly false / Definitely false

**१६. म मेरो परिवारको लागि बोझ महसुस गर्छु।***16. I feel like a burden on my family.*- पूर्ण सत्य / प्रायःसत्य / थाहा छैन / प्रायः गलत / पूर्ण गलत / Definitely true / Mostly true / Don't know / Mostly false / Definitely false

## खण्ड ७: लक्षणहरू (विगत ४ हप्ताभित्र) / Section 7: Symptoms (Past 4 Weeks)

**१७. मांसपेशी दुखाइ***17. Muscle soreness*- बिल्कुलै छैन / अलिकति / मध्यम / धेरै / अत्यन्तै / Not at all / A little bit / Moderately / Quite a bit / Extremely

**१८. छाती दुखाइ***18. Chest pain*- बिल्कुलै छैन / अलिकति / मध्यम / धेरै / अत्यन्तै / Not at all / A little bit / Moderately / Quite a bit / Extremely

**१९. ऐंठन (क्र्याम्प्स)***19. Cramps*- बिल्कुलै छैन / अलिकति / मध्यम / धेरै / अत्यन्तै / Not at all / A little bit / Moderately / Quite a bit / Extremely

**२०. छालामा खुजली***20. Itchy skin*- बिल्कुलै छैन / अलिकति / मध्यम / धेरै / अत्यन्तै / Not at all / A little bit / Moderately / Quite a bit / Extremely

**२१. सुख्खा छाला***21. Dry skin*- बिल्कुलै छैन / अलिकति / मध्यम / धेरै / अत्यन्तै / Not at all / A little bit / Moderately / Quite a bit / Extremely

**२२. सास फेर्न गाह्रो***22. Shortness of breath*- बिल्कुलै छैन / अलिकति / मध्यम / धेरै / अत्यन्तै / Not at all / A little bit / Moderately / Quite a bit / Extremely

**२३. टाउको घुम्ने वा बेहोस हुने***23. Dizziness or faintness*- बिल्कुलै छैन / अलिकति / मध्यम / धेरै / अत्यन्तै / Not at all / A little bit / Moderately / Quite a bit / Extremely

**२४. भोक नलाग्ने***24. Lack of appetite*- बिल्कुलै छैन / अलिकति / मध्यम / धेरै / अत्यन्तै / Not at all / A little bit / Moderately / Quite a bit / Extremely

**२५. थकाइ वा शक्तिहीनता***25. Washed out or drained*- बिल्कुलै छैन / अलिकति / मध्यम / धेरै / अत्यन्तै / Not at all / A little bit / Moderately / Quite a bit / Extremely

**२६. हात वा खुट्टामा सुन्निने***26. Numbness in hands or feet*- बिल्कुलै छैन / अलिकति / मध्यम / धेरै / अत्यन्तै / Not at all / A little bit / Moderately / Quite a bit / Extremely

**२७. वाकवाकी वा पेट खराब***27. Nausea or upset stomach*- बिल्कुलै छैन / अलिकति / मध्यम / धेरै / अत्यन्तै / Not at all / A little bit / Moderately / Quite a bit / Extremely

**२८^a^. (हेमो डायलाइसिस बिरामीका लागि) एक्सेस साइटमा समस्या***28^a^. Problems with your access site (for hemodialysis patients)*- बिल्कुलै छैन / अलिकति / मध्यम / धेरै / अत्यन्तै / Not at all / A little bit / Moderately / Quite a bit / Extremely

**२८^b^. (पेरिटोनियल डायलाइसिस बिरामीका लागि) क्याथेटर साइटमा समस्या***28^b^. Problems with catheter site (for peritoneal dialysis patients)*- बिल्कुलै छैन / अलिकति / मध्यम / धेरै / अत्यन्तै / Not at all / A little bit / Moderately / Quite a bit / Extremely

## खण्ड ८: दैनिक जीवनमा असर / Section 8: Effects on Daily Life

**२९. तरल पदार्थ खान प्रतिबन्ध***29. Restriction of fluid intake*- बिल्कुलै छैन / अलिकति / मध्यम / धेरै / अत्यन्तै / Not at all / A little bit / Moderately / Quite a bit / Extremely

**३०. खानामा प्रतिबन्ध***30. Restriction of diet*- बिल्कुलै छैन / अलिकति / मध्यम / धेरै / अत्यन्तै / Not at all / A little bit / Moderately / Quite a bit / Extremely

**३१. घर वरिपरिको काम गर्ने क्षमता***31. Ability to do household chores*- बिल्कुलै छैन / अलिकति / मध्यम / धेरै / अत्यन्तै / Not at all / A little bit / Moderately / Quite a bit / Extremely

**३२. यात्रा गर्ने क्षमता***32. Ability to travel*- बिल्कुलै छैन / अलिकति / मध्यम / धेरै / अत्यन्तै / Not at all / A little bit / Moderately / Quite a bit / Extremely

**३३. चिकित्सक वा स्वास्थ्यकर्मीमा निर्भर हुनु***33. Dependence on doctors or other health professionals*- बिल्कुलै छैन / अलिकति / मध्यम / धेरै / अत्यन्तै / Not at all / A little bit / Moderately / Quite a bit / Extremely

**३४. मृगौला रोगका कारण तनाव वा चिन्ता***34. Worry or concern due to kidney disease*- बिल्कुलै छैन / अलिकति / मध्यम / धेरै / अत्यन्तै / Not at all / A little bit / Moderately / Quite a bit / Extremely

**३५. यौन जीवन***35. Sexual life*- बिल्कुलै छैन / अलिकति / मध्यम / धेरै / अत्यन्तै / Not at all / A little bit / Moderately / Quite a bit / Extremely

**३६. व्यक्तिगत देखावट***3०. Personal appearance*- बिल्कुलै छैन / अलिकति / मध्यम / धेरै / अत्यन्तै / Not at all / A little bit / Moderately / Quite a bit / Extremely
